# Migration of ions near charged surface

**DOI:** 10.1371/journal.pone.0250343

**Published:** 2021-04-28

**Authors:** Kiwoong Kim

**Affiliations:** Department of Mechanical Engineering, Hannam University, Daejeon, Republic of Korea; Martin-Luther-Universitat Halle-Wittenberg, GERMANY

## Abstract

Detailed understanding of ionic behavior in the region near a charged surface is important for the enhancement of water filtration mechanisms. In this study, a highly charged membrane is hypothesized to form an ion depletion zone (IDZ) without an external power supply. The formation of IDZ was experimentally investigated using membranes with varying surface zeta potential (SZP) values to confirm the hypothesis. The surface zeta potential of the charged membrane was controlled by layer-by-layer deposition method. Our results indicate that indicated that the fluorescent intensity near the charged surface becomes weaker with an increased absolute magnitude of SZP. Ionic surfactants enhance the formation of IDZ by increasing SZP magnitude, and by forming a secondary filtration layer. These findings provide information that is helpful in understanding the ionic behavior near the highly charged surface. In addition, the information provided by the findings would be helpful in fabricating a small-scale water filtration device.

## Introduction

The growing global population, causing a high demand for water that outpace supply, and water pollution due to industrialization, have led to water shortage and, driven the need for water purification [1]. In addition, low-quality water and poor sanitation cause approximately 1.7 million deaths annually [2]. Therefore, the development of an enhanced water purification technology is essential for guaranteed adequate and safe drinking water supply.

While plants need water as an essential element for various metabolic activities [3], some also offer an interesting advantage of water filtration ability [4–6]. However, this advantage has not been fully utilized, because the underlying biophysical features of the water filtration mechanisms remain unclear. Mangroves growing in coastal saline or brackish water are halophytes which survive under saline condition owing to their saline water filtration ability through inherent hydraulic survival strategies [7, 8]. In our previous study, we investigated water filtration distinctive features of the mangroves, among them, the surface zeta potential (SZP) of root which we found to be approximately −91.4 mV. This SZP value is noticeably higher than those of conventional water filtration membranes [9]. As one of the survival strategies of the mangroves, the highly negatively charged surface seems to contribute to salt filtration from saline water. However, the validity of this hypothesis has not been examined in detail yet.

Surface properties, such as wettability, roughness and charge play significant roles in a number of membrane-based water management techniques [10–14]. Electrostatic surface charge is a dominant factor in water filtration owing to the importance of electrostatic interactions in the rejection of ionic molecules [15]. Chemical treatments [16, 17], pH adjustment [18], and ionic surfactants [19, 20] have previously been utilized to control SZP. In this study, to understand the underlying mechanism of ion rejection by electrostatic interactions, we experimentally investigated the influence of SZP on the formation of the ion depletion zone (IDZ). Several membranes with different SZP values were tested and quantitative comparison was done.

**Fig 1:**
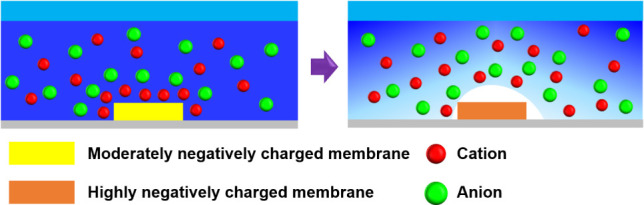
Formation of IDZ according to SZP of a membrane. Schematics illustrating IDZ formation around the membrane. Cations (red circle) are attracted toward a negatively charged surface, whereas anions (green circle) are repelled from the surface of a moderately charged membrane (yellow square) when the membrane is exposed to an ionic solution. Ion depletion zone is rarely formed in this case. However, when a highly negatively charged membrane (orange square) is exposed to the ionic solution, formation of IDZ (white area) is enhanced.

As shown in Fig 1, in this study, IDZ was rarely observed in membranes with a moderately charged surface. However, as the membrane SZP became highly negative, IDZ was formed in the region near the charged surface. Thus, ion rejection was mainly governed by electrostatic interactions, and a highly negatively charged membrane was essential for water filtration without external power supply.

## Materials and methods

### Preparation of experimental model

**Fig 2:**
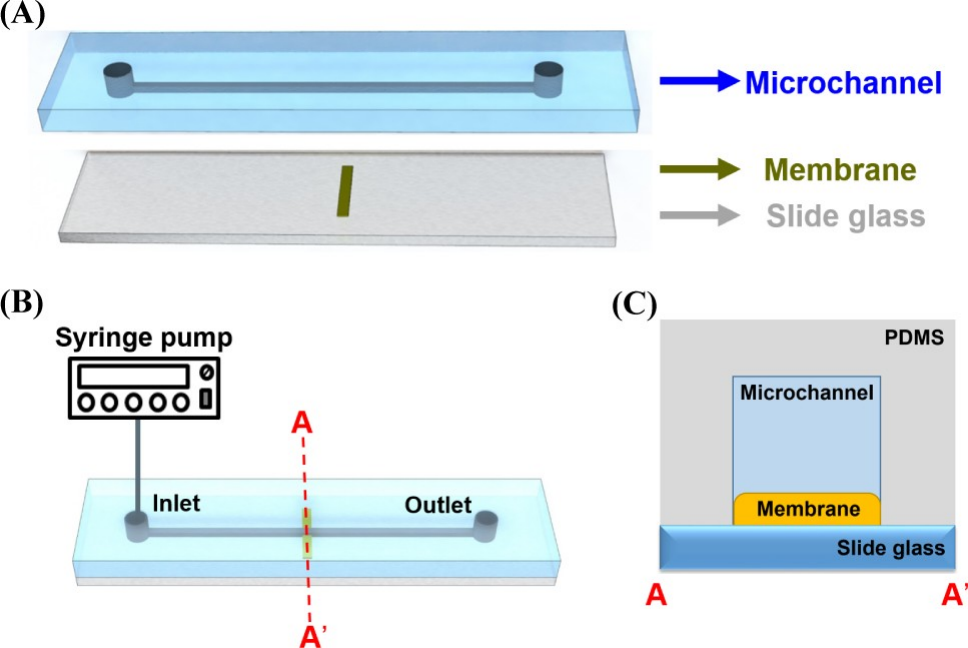
A schematic of the experimental setup. (A) Two microchannels with a width of 500 μm in width and depths of 55 and 10 μm were fabricated. Three membranes with different SZP values were inserted between a slide glass and the PDMS channel. (B) The PDMS channel and the slide glass were bonded using O_2_ plasma etching. The feed solution containing an ionic solution and fluorescent dyes were supplied to the inlet, and behaviors of fluorescent dyes were observed using an inverted microscope. (C) A cross-sectional view of the experimental setup.

Microchannel molds were printed directly (Prototech, Seoul, Korea) from computer-aided design files. A base-to-catalyst mixture (10% w/w) of silicone elastomer (PDMS, Sylgard 184, Dow Corning, Midland, MI, USA) was poured onto the mold, desiccated, and cured at 80°C for 1 h. The device was detached from the mold. Two microchannels were prepared to visualize IDZ formation near the membranes (Fig 2A). Their physical dimensions (length × width × depth) were 25,000 μm × 500 μm × 55 μm and 25,000 μm × 500 μm × 10 μm. Three different membranes, 500 μm wide, 10 μm thick, and 100 nm pore size were installed at the center of a slide glass, and bonded using oxygen plasma treatment. Formation of ion depletion zone formation is quite affected by the flow rate because most of the ions are rejected near the charged surface of the membrane by the Donnan exclusion principle. The electrostatic interaction between the charged surface and ions is dominant, so convection rate should be less than diffusion rate. In this view point, flow rate of feed solution was applied as 0.1 μl/min. (Fig 2B).

In the present study, the effect of SZP values of a charged surface on IDZ formation without external power supply was investigated. Three membranes with different SZP were tested (Table 1). A layer-by-layer deposition treatment was employed to control the membranes SZP. Poly-allylamine hydrochloride [cationic polyelectrolyte (PAH), Sigma–Aldrich, Korea] and polystyrene sulfonate hydrochloride [anionic polyelectrolyte (PSS), Sigma–Aldrich, Korea] were deposited on a polyethylene-terephthalate-based membrane (PET, Sterlitech Co., Kent, USA). The thickness and pore size of tested membrane were 10 μm and 100 nm, respectively. The detailed procedures to fabricate a charged membrane and to measure of SZP of the membrane are appended in the S1 File and referred paper [21].

**Table 1:** Characteristics of the membranes tested in this study.

Case	Feature	Surface zeta potential
**A**	PAH-treated PET	-36.2 ± 4.4 mV
**B**	Carboxylation PET	-62.5 ± 3.8 mV
**C**	PAH–PSS-treated PET	-97.5 ± 4.3 mV

### Visualization of IDZ

Rhodamin 6G [positively charged fluorescent dye (PFD), Sigma–Aldrich, Korea], Alexa 488 [negatively charged fluorescent dye (NFD), Invitrogen, USA], and phosphate-buffered saline (PBS) solution were used to visualize ionic behavior near the charged surface. The excitation and emission peak of the PFD is 525 nm and 548 nm, respectively. The excitation and emission peak of the NFD is 493 nm and 519 nm, respectively. Sodium dodecyl sulfate (SDS, anionic surfactant, Sigma–Aldrich, Korea) was used to change the SZP magnitude. Ion depletion zone was visualized with an inverted fluorescent microscope (Axiovert 200, Zeiss) attached to a charge-coupled device camera (Q42286, Q-IMAGING, Canada). The recorded images were analyzed using the ImageJ program (National Institute of Health, USA). The fluorescent intensity values were normalized to compare the average fluorescence intensity in the right side of the membrane surface and that in the left side (S1 Fig in S1 File). The fluorescence intensity was calculated from five different lines within the margin of error of 5%.

## Results and discussion

### Intensity variations of IDZ

Fig 2 shows the experimental setup for this study. The depths of the two fabricated microchannels were 55 and 10 μm. The membrane was set on a slide glass in advance and then the PDMS channel and the slide glass were bonded using O_2_ plasma etching (Fig 2A) this way the membrane was located at the microchannel bottom (Fig 2B). A feed solution(pH 7.2) that contained 0.1 mM of PFD, 0.1 mM of NFD, and 0.1 mM of PBS passed through the membrane at a flow rate of 0.1 μl/min. The ionic behaviors of fluorescent dyes were observed using an inverted fluorescent microscope (Zeiss Axiovert 200, Germany).

**Fig 3:**
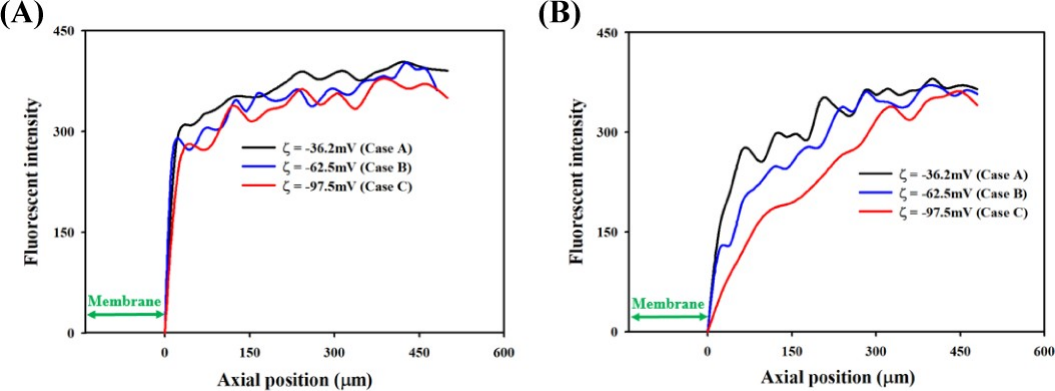
Variations of IDZ intensity formed near the charged membrane. The fluorescent intensity of sum of PFD and NFD was analyzed using three membranes with different SZP Magnitude. The fluorescent profile was calculated using the membrane marked by the green arrow. Formation of IDZ means ion depleted solution compared to feed solution is obtained. This result indicated the concentration distribution along the microchannels (A) a 55 μm and (B) a 10 μm.

The investigation of the formation of IDZ using two microchannels with depths of 55 and 10 μm was conducted (S2 Fig in S1 File). The fluorescent intensities of PFD and NFD were quantitatively analyzed at varying distances from the membrane surface. Fig 3 shows the fluorescence intensity of both dyes. In the microchannel with a 55 μm depth, the fluorescent intensity rapidly increased after the membrane (Fig 3A). A similar tendency occurred with increased membrane SZP magnitude. The fluorescent intensities rapidly increased up to the axial position of approximately 50 μm. Thereafter, they gradually increased with insignificant fluctuations. Ion depletion zone formation was rarely observed in these experimental conditions (S3A Fig in S1 File). In the microchannel with a 10 μm depth, IDZ was clearly observed (S3B Fig in S1 File). The fluorescent intensities were weaker than those of the microchannel with a 55 μm depth (Fig 3B). In the cases of A and B, the fluorescent intensities steeply increased up to the axial position of approximately 65 μm. Thereafter, the increase was gradual. For the highly negatively charged membrane with SZP value of −97.5 mV (Case C), the fluorescent intensity increased from 0 to 340 up to the axial position of approximately 350 μm. These findings indicated that the formation of IDZ along the axial was largely affected by the channel depth and membrane SZP values. The gradient in strength depletion along the vertical direction affected IDZ formation because the charged membrane has a limited capacity to form a three-dimensional IDZ [22]. These results indicate that the shorter microchannel positively impacts the formation of IDZ along the axial direction.

For a better understanding of SZP effects, each fluorescent intensities of PFD and NFD in the microchannel with a 10 μm depth were compared. For quantitative comparison, the average values of PFD and NFD within 150 μm from the right-side surface of the membrane were normalized by the average value of the initial fluorescent intensity within 150 μm from the left-side surface of the membrane (S1 Fig in S1 File). Because the difference in fluorescence intensity according to the surface charge of the tested membranes was the largest at a point 150 um apart (Fig 3).

**Fig 4:**
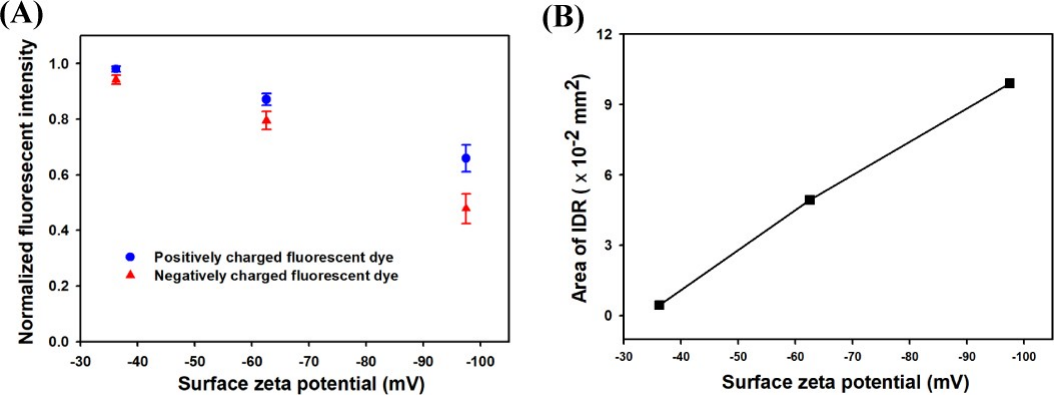
Formation of IDZ near the charged membranes. (A) The normalized fluorescent intensities of PFD and NFD were measured, respectively. (B) The IDZ area defined as the region whose intensity was lower than 220 was calculated.

Fig 4A shows that the normalized fluorescent intensity of PFD was 0.98 after passing through a membrane with SZP of −36.2 mV (Case A). The normalized fluorescent intensity of PFD gradually decreased from 0.87 to 0.66 as SZP became more negative from −62.5 mV (Case B) to −97.5 mV (Case C). The normalized fluorescent intensity of NFD was 0.94 after the feed solution passed through the membranes of Case A. However, the value decreased from 0.79 to 0.48 after the feed solution passed through the membranes of Cases B and C. Therefore, the rate of decrease in NFD intensity was stronger than that of PFD because indicating that the highly charged membrane was more efficient in excluding co-ions from the membrane surface.

The counter-ion concentration was generally higher in the region near the membrane surface than in the bulk solution, whereas the co-ion concentration was lower in the same region due to the membrane surface charge. These findings confirm that the electrostatic force attracted counter-ions but repelled co-ions from the charged membrane surface [23]. Thus, the concentration of NFD was smaller than that of PFD. As SZP magnitude increased, counter-ions became more dominant than co-ions in the region near the membrane surface. A potential difference, which was called Donnan potential, was established on the membrane surface. The electroneutrality condition prevented the independent migration of counter-ions and co-ions [24]. As a result, the rejection of counter-ions and co-ions led to the formation of IDZ near the charged membrane surface.

The variations of IDZ according to the SZP magnitude were determined by calculating IDZ area (Fig 4B). Ion depletion zone formation area was defined as the region whose intensity is lower than 220. The visualized images of formation of IDZ was loaded with the Image J program, and the area of the IDZ was calculated by counting the number of pixels whose intensity is less than 220 in the image. As shown in Fig 3, if you look at the intensity distribution of the image, it exists between 0 and 440. So, we decided to use 220, which is half the value. Ion depletion zone formation area was 0.45 × 10^−2^ mm^2^ after passing the membrane with SZP of −36.2 mV (Case A). Ion depletion zone formation As SZP was decreased from −62.5 mV (Case B) to −97.5 mV, IDZ area significantly increased from 4.93 × 10^−2^ to 9.90 × 10^−2^ mm^2^ (Case C). Based on the present study experimental results, we confirmed that the formation of IDZ near the charged membrane could be enhanced by a highly negatively charged surface.

### Ionic surfactant effect

**Fig 5:**
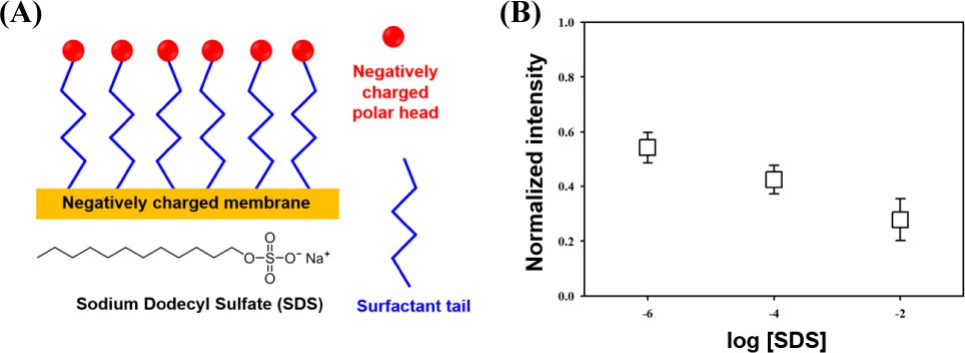
Effect of ionic surfactant on normalized intensity. (A) A schematic of SDS molecules adsorption on the negatively charged membrane surface. (B) Variation in normalized fluorescent intensity according to SDS concentration.

An ionic surfactant was added to the feed solution to enhance the formation of IDZ near the charged membrane. Fig 5A is a schematic of the surface treatment process through a charged membrane using SDS. The membrane which has -97.5 ± 4.3 mV in surface zeta potential (Case C) and 10 um in thickness was tested. As SZP of the membrane was affected by the solution pH, the negatively charged sulfate functional groups of the SDS made the membrane surface more negatively charged [20]. In this study, the SDS effect was investigated at the concentration of 0.001 mM, 0.1 mM and 10 mM (pH 7.2). When SDS was added to the feed solution, there was a hydrophobic interaction between the SDS molecules and the negatively charged membrane surface. As a result, the SDS tails were attached on the membrane surface, and SZP of the membrane became highly negative due to the existence of a number of charged polar heads. Fig 5B shows the variation in normalized fluorescent intensity in the microchannel with a 10 μm depth, according to SDS concentration. As the SDS concentration increased, the normalized fluorescent intensity decreased from 0.54 to 0.28. Therefore, membrane SZP was an important factor in IDZ formation. The ionic surfactant did not only enhance SZP, but induced a secondary filtration layer on the charged membrane surface. As a result, the formation of IDZ near the charged membrane was enhanced. Although the concentration of the ionic solution tested in this experiment was relatively low, the results would be useful in understanding the influence of SZP in enhancing the formation of IDZ. The further studies are planned to characterize the influence of SDS for the formation of IDZ.

## Conclusions

In the present study, the relationship between IDZ formation and SZP values of a charged membrane was. Anionic and cationic polyelectrolytes were deposited on the membrane surface to control the membrane SZP. The formation of IDZ on membranes and the ionic behavior were observed. When the charged membranes contacted an ionic solution, a concentration gradient was induced, normal to the membrane surface. Ion depletion zone formation was formed near the charged surface to satisfy the electroneutrality (Fig 1). Case C had the most negatively charged surface; consequently, immense IDZ formation, and lower intensity than that of the other cases with smaller SZP. The difference in normalized fluorescent intensities between PFD and NFD was attributed to the enrichment of membrane surface with counter-ions and the depletion of co-ions due to electrostatic force. As a result, the electroneutrality was regenerated in the region slightly far from the charged membrane surface by the aid of the well-known Donnan exclusion mechanism [25]. The formation of IDZ along the flow direction could be enhanced by decreasing the microchannel aspect ratio (depth/width).

In the present study, negatively charged membrane surface became highly negative by adding an ionic surfactant to the feed solution, these results suggest that IDZ could be enhanced by the formation of an additional filtration layer by the hydrocarbon chains and charged polar heads of the ionic surfactant. This expanded conformation might enhance the rejection rate of the ionic molecules. Ion depletion zone formation area was increased from 9.90 × 10^−2^ to 17.56 × 10^−2^ mm^2^ when 0.01 mM of SDS was added into the feed solution. Therefore, the highly negatively charged membrane can be effectively utilized to form IDZ without external supply electricity.

Mangrove roots exhibit a considerably high ion rejection rate and stable water permeability, although the sap flow rate is relatively low. In our previous study, we found that the water filtration ability of the mangrove roots is attributed to the highly negatively charged surface [7]. Similarly, the present study results demonstrated that the highly negatively charged membrane not only enhances IDZ formation without external power supply but also enhances IDZ formation near the charged surface. Our findings provide basic information on the effect of SZP on ionic transport and would be helpful in understanding the underlying water filtration mechanism of mangrove roots. In addition, based on these findings, small-scale and power-free water filtration devices can be developed by utilizing the proposed enhancement technique for the development of improved water purification methods.

## Supporting information

S1 File (DOCX)
