# Supplementary material for: Migration of ions near charged surface
Source: PLoS One. 2021 Apr 28;16(4):e0250343. doi: 10.1371/journal.pone.0250343 (PMC8081195; doi:10.1371/journal.pone.0250343)
Supplement: S1 File — (DOCX) [file pone.0250343.s001.docx]

**Migration of ions near charged surface**

Kiwoong Kim*

Department of Mechanical Engineering, Hannam University, Daejeon 34430, Korea.

*E-mail: kwkim@hnu.kr


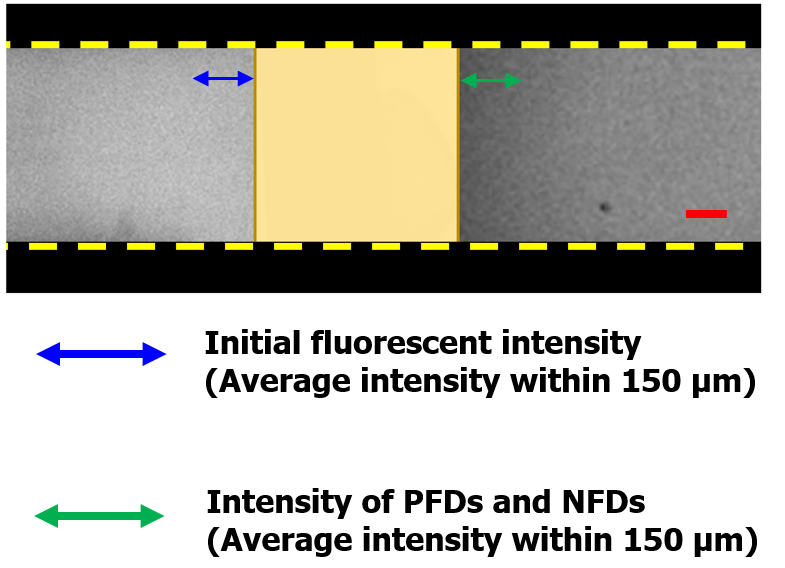


**S1 Fig. Spatial distributions of the fluorescent intensities of the PFDs and NFDs.** Averaged fluorescent intensities of the PFDs and NFDs within 150 μm (marked by green arrow) were normalized by the averaged initial fluorescent intensity within150 μm (marked by blue arrow). Scale bar: 100 μm.


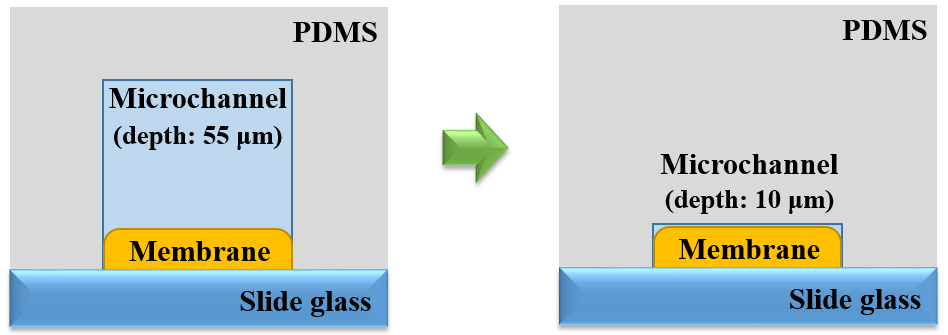


**S2 Fig. Experimental models.** Two microchannels with depths of 55 and 10 μm.


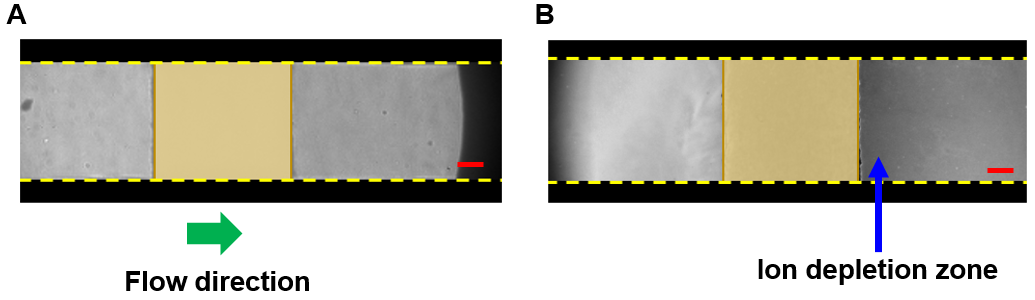


**S3 Fig. Visualization of fluorescent dyes.** (A) Fluorescent image of dye in the microchannel with a 55 μm depth. The patterns of intensity distributions before and after the charged membrane were similar. (B) In the microchannel with a 10 μm depth, the IDZ marked by blue arrow was clearly observed. Scale bars: 100 μm.

**Membrane preparations**

The layer-by-layer deposition method was employed to control membrane SZP. The PET membrane was rinsed with deionized water, methanol, and hexane for 2 h and then immersed in a1 M NaOH aqueous solution for 20 min. As a result, a carboxylation PET membrane was fabricated. The carboxylate-terminated membranes were immersed in 1 mg/mL PAH solution to fabricate the PAH membrane. Thereafter, the PAH membrane was dipped in 1 mg/mL PSS solution to make a PAH−PSS membrane. PAH and PSS were firmly deposited on the PET-based membrane by electrostatic force.

**Measurement of surface zeta potential of charged membrane**

The SZP value of the fabricated membranes was measured using a Zetasizer Nano (Malvern Co., Worcestershire, UK). Polystyrene latex beads (Malvern Co., Worcestershire, UK) with mean charge of −42 ± 4.2 mV were used as tracer particles. The polystyrene latex standard particles were seeded in an aqueous buffer solution of pH 9.

The surface zeta potential measurement cell (Malvern Co., Worcestershire, UK) which consist of a height adjustable sample holder and two electrodes was used. The cell is in a dip cell format with a rotating top attached to a screw thread allowing the sample height, and thus the displacement, to be altered. The sample is attached to the cell and submersed in a medium containing tracer particles. The application of an electric field via the electrodes will initiate electrophoresis of the particles. Close to the sample surface, electro-osmosis will also be established. The mobility of the particles in the system will be a balance between these two forces. The closer the tracer particle to the sample surface the greater the significance of the electro-osmosis on the particle mobility. As the particle distance from the surface increases, the effect of the electro-osmosis will decrease to the point at which particle movement is entirely dependent on electrophoresis. It is clear that the measured electrophoretic mobility will therefore vary as a function of distance from the sample surface. By plotting the reported mobility, or zeta-potential, as a function of displacement from the surface, the relationship can be extrapolated back to the intercept, or zero displacement. Since the measured zeta potentials always include a component of the electrophoretic motion, the measured intercept will necessarily include this component which must be accounted for. In fact, the wall potential is defined by equation :

ξwall = -intercept + ξparticle(1)

The SZP of the carboxylation PET membrane was −62.5 ± 3.8 mV; that of the PAH membrane, on which the positively charged layer was deposited, was approximately −36.2 ± 4.4 mV; that of the PAH–PSS membrane, which had negatively charged electrolytes on the surface, was approximately −97.5 ± 4.3 mV.
